# High-Throughput GoMiner, an 'industrial-strength' integrative gene ontology tool for interpretation of multiple-microarray experiments, with application to studies of Common Variable Immune Deficiency (CVID)

**DOI:** 10.1186/1471-2105-6-168

**Published:** 2005-07-05

**Authors:** Barry R Zeeberg, Haiying Qin, Sudarshan Narasimhan, Margot Sunshine, Hong Cao, David W Kane, Mark Reimers, Robert M Stephens, David Bryant, Stanley K Burt, Eldad Elnekave, Danielle M Hari, Thomas A Wynn, Charlotte Cunningham-Rundles, Donn M Stewart, David Nelson, John N Weinstein

**Affiliations:** 1 Genomics and Bioinformatics Group, Laboratory of Molecular Pharmacology, National Cancer Institute, National Institutes of Health, Bethesda, MD 20892, USA; 2 Metabolism Branch, National Cancer Institute, National Institutes of Health, Bethesda, MD 20892, USA; 3 SRA International, 4300 Fair Lakes CT, Fairfax, VA 22033, USA; 4 Advanced Biomedical Computing Center, National Cancer Institute at Frederick, SAIC Frederick, PO Box B, Frederick, MD, 21702, USA; 5 Laboratory of Parasitic Disease, National Institute of Allergy and Infectious Diseases, National Institutes of Health, Bethesda, MD 20892, USA; 6 The Mount Sinai Medical Center, 1425 Madison Avenue, New York, NY 10029, USA

## Abstract

**Background:**

We previously developed GoMiner, an application that organizes lists of 'interesting' genes (for example, under-and overexpressed genes from a microarray experiment) for biological interpretation in the context of the Gene Ontology. The original version of GoMiner was oriented toward visualization and interpretation of the results from a single microarray (or other high-throughput experimental platform), using a graphical user interface. Although that version can be used to examine the results from a number of microarrays one at a time, that is a rather tedious task, and original GoMiner includes no apparatus for obtaining a global picture of results from an experiment that consists of multiple microarrays. We wanted to provide a computational resource that automates the analysis of multiple microarrays and then integrates the results across all of them in useful exportable output files and visualizations.

**Results:**

We now introduce a new tool, High-Throughput GoMiner, that has those capabilities and a number of others: It (i) efficiently performs the computationally-intensive task of automated batch processing of an arbitrary number of microarrays, (ii) produces a human-or computer-readable report that rank-orders the multiple microarray results according to the number of significant GO categories, (iii) integrates the multiple microarray results by providing organized, global clustered image map visualizations of the relationships of significant GO categories, (iv) provides a fast form of 'false discovery rate' multiple comparisons calculation, and (v) provides annotations and visualizations for relating transcription factor binding sites to genes and GO categories.

**Conclusion:**

High-Throughput GoMiner achieves the desired goal of providing a computational resource that automates the analysis of multiple microarrays and integrates results across all of the microarrays. For illustration, we show an application of this new tool to the interpretation of altered gene expression patterns in Common Variable Immune Deficiency (CVID). High-Throughput GoMiner will be useful in a wide range of applications, including the study of time-courses, evaluation of multiple drug treatments, comparison of multiple gene knock-outs or knock-downs, and screening of large numbers of chemical derivatives generated from a promising lead compound.

## Background

The original version of GoMiner [1,2] was oriented toward visualization and interpretation of the results from a single microarray (or other high-throughput experimental platform), using a graphical user interface (GUI). Although the GUI can be used to examine the results from a number of microarrays one at a time, that is a rather tedious task, and there is no apparatus for obtaining a global picture of results from an experiment that consists of multiple microarrays:

• Suppose, for example, that combinatorial chemistry were used to generate a large number of derivatives of a lead compound. If microarrays were used to monitor the efficacy of those derivatives, then it is likely that none, or at most a few, of the microarrays would be interesting. It would be a thankless task to use the GUI to analyze and interpret the large number of uninformative microarrays. It would make much more sense to apply an automated batch procedure to generate a report that highlighted the interesting microarrays and then to examine just those in the GUI.

• As another example, suppose that a series of microarrays were used to generate a time-course. One would want to obtain a high-level, global picture of the relationships of the categories that were significant at different time points – for instance, to differentiate phases of a disease process or to explore the temporal sequence of events consequent to treatment with a drug.

High-Throughput GoMiner performs those tasks. As a tool for investigators with large sets of results, it complements and extends the GUI version's analysis and visualization capabilities. Both the command line and web application interfaces of High-Throughput GoMiner are freely available to all users [3]. To our knowledge, this is the first resource that integrates information and illuminates patterns from multiple microarrays in relationship to the Gene Ontology.

In the original GoMiner article [1,2], we noted that the Fisher's exact p-values require adjustment to account for the multiple comparisons problem. We proposed a resampling approach that would avoid major drawbacks of the Bonferroni correction (see, for example [4]) – the assumption of independence of categories and the likelihood of rejecting too many *true *positives. To provide a more balanced solution to the problem, we have now implemented a fast 'false discovery rate' (FDR) approach in High-Throughput GoMiner.

Another noteworthy feature of High-Throughput GoMiner is the integration of transcription factor binding site information with GO categorization and gene expression data so that the user can explore regulatory relationships. Although each of these types of information has been used alone previously, our approach of integrating them provides a powerful, novel analysis tool.

In addition to the functionality that it provides, High-Throughput GoMiner also serves as a model for integration of the command line interface of the original GoMiner into other applications. The command-line interface permits platform-independent integration of GoMiner's functionality into any data processing stream without modification of the GoMiner source code.

### Overview of High-Throughput GoMiner

Throughout the text of this article, we will focus for concreteness of terminology on use of High-Throughput GoMiner for gene expression microarrays, but the range of application is much broader; it can be used for any high-throughput data set in which genes or proteins are flagged as 'interesting' for whatever reason, either as the result of a real experiment or a 'conceptual' *in silico *experiment.

The program requires two kinds of input: a list of the total set of genes on the microarray and a set of 'changed-gene' files. Each changed-gene file contains a subset of genes that the user considers interesting in the experiment (for example, genes that are under-or over-expressed). The formats of the files are the same as those required for the original GoMiner. High-Throughput GoMiner creates two types of output. The first is a set of reports and data files integrating the results from all of the microarrays. The second is a set of subdirectories, each of which contains results files for one of the microarrays.

Two especially noteworthy features of the data processing stream are (i) the implementation of a fast, efficient solution to the multiple testing problem (see sections on 'Computational Efficiency of High-Throughput GoMiner' and 'The Multiple Comparisons Problem in High-Throughput GoMiner: Estimating FDRs') and (ii) the integration of results from multiple microarrays.

There are a large number of output files. Some of them focus on the results from a particular microarray; others integrate the results from all of the microarrays. Both types can be used as input to tools such as Excel or CIMminer [5,6] to provide an integrative visualization of the results of one or all microarrays in the study. The companion web site [3] contains a detailed description of the input and output files.

### Significant advances relative to the original version of GoMiner

High-Throughput GoMiner provides significant advances relative to the original GoMiner. Those advances are manifested in both scientific value-added and usability.

### Scientific value-added

#### 1. Integration across multiple microarray experiments: CIMs

The primary scientific value-added in High-Throughput GoMiner relative to the original GoMiner is *integration *of the results across multiple related microarrays. That type of integration is particularly useful when the set of changed-gene files represents a time course, such as progression of a disease, response to a drug, or development of an organism. The integration can also be useful in pharmaceutical discovery and development – for example in the parallel testing of many combinatorial chemistry products against cells or organisms when microarrays are used to provide multiplexed assay end-points. The diagnostics in High-Throughput GoMiner can indicate which compounds appear to be related in their activity to which GO categories.

Visual integration of results is achieved by producing CIMminer [6] input files. CIMminer is our program package for computing and displaying clustered image maps (CIMs). We introduced CIMs [5], also called clustered 'heat maps', in the mid-1990's, and they have since become the ubiquitous summary graphic for high-throughput 'postgenomic' data, for example from microarray experiments. We have extended the CIM paradigm to permit visualization of significant GO categories integrated across multiple microarrays.

#### 2. Integration across multiple microarray experiments: category|gene-disease export files

Another form of integration is provided by a set of files each of which contains a matrix whose rows are category|changed gene pairs and whose columns are names of changed-gene files. When the names of changed-gene files are diseases to be compared, we refer to the output as the 'category|gene-disease' set. That set of output files is designed for analysis by the CIMminer program package. Clinical collaborators have found that the resulting CIMs greatly facilitate analysis of genes with altered expression and the interpretation of significant GO categories across related disease phenotypes.

#### 3. CIMs of genes with altered expression versus significant GO categories

High-Throughput GoMiner generates a set of files for producing CIMs of genes with altered expression *versus *significant GO categories. Those CIMs facilitate determination of the relatedness of significant GO categories as defined by the degree of sharing of genes. The importance of that capability can be demonstrated by two canonical examples:

• Several GO categories can be combined into a single cluster if they contain essentially the same set of changed genes. Combining multiple categories in that manner brings about a simplification since the user can think in terms of a smaller number of clusters of related categories rather than in terms of a larger number of individual categories. That procedure is especially important because the parent-child structure of GO can result in a number of statistically significant categories that may contain nearly redundant sets of changed genes.

• Apparently unrelated GO categories might in fact be connected by containing changed genes in common. Such 'cross talk' can often explain apparently surprising instances in which unexpected GO categories achieve statistical significance.

#### 4. Annotation of genes and GO categories with transcription factor binding site information

High-Throughput GoMiner generates automated annotation of genes and GO categories with transcription factor binding site information. For instance, one output in this set is a file whose columns are significant GO categories and whose rows are transcription factor binding sites for the genes within each category. This enhancement was motivated by the intense interest in inferring genomic regulatory networks from the results of microarray experiments.

### Improvements in usability

#### 1. Automated batch processing of an arbitrary number of changed-gene files

The original GoMiner requires a substantial number of manual operations to analyze a changed-gene file. That process is feasible for analysis of one or two files but becomes tedious, time-consuming, and error-prone when more files are to be analyzed. In a high-throughput context, there may be dozens or even hundreds of files to analyze for a study. It would be virtually impossible to use the original GoMiner to analyze that number of files. A human operator would almost inevitably introduce errors in the I/O, analysis, and book-keeping phases. Furthermore, in a large set of files, there may be only one or two that produce interesting results. Manual analysis of a hundred files to identify one or two files of interest would not be cost effective. In contrast, High-Throughput GoMiner automates both the analysis of any number of files and the selection of those files that are likely to be worth follow-up.

#### 2. Automated report generation

High-Throughput GoMiner generates a report that summarizes the results from all microarrays, with the best ones – those with the largest number of significantly enriched categories – presented at the top of a sorted list. The user (or the computer if further downstream processing is to be performed) can focus principally on the top entries in the report.

The complete analysis process is documented and can be reviewed if a question arises later. If the original GoMiner had been used and a question arose about whether file number 57 was processed correctly, or whether the results from file number 57 were recorded correctly, many manual operations would need to be repeated. In contrast, High-Throughput GoMiner would require only a few moments. Accurate summary reports and a well-defined and reproducible directory structure make all of the results immediately available. The output files and directory structure permit user-friendly access to high-level information that characterizes all of the changed-gene files as well as to detailed information about the results for any particular changed-gene file. The output of High-Throughput GoMiner is essentially self-documenting. With the original GoMiner, manual record-keeping is a considerable burden; the automated record-keeping in High-Throughput GoMiner is a major asset.

#### 3. Elimination of relatively slow access to our database server

Another important usability issue encountered by a number of users of the original GoMiner is relatively slow access to our database server. That issue can arise because of the high overhead of performing successive database accesses *via *the internet. The problem is not fatal, but in practice it can limit the number of changed-gene files that a user is able to process manually in a reasonable amount of time. To overcome that limitation, the original GoMiner web site provides support for the user with IT skills who wants to install a local version of the database. Unfortunately, such in-house IT support may be unavailable to many clinicians and biologist. The web version of High-Throughput GoMiner eliminates that problem because it processes the web-based queries on our server using the database that is local to our server.

### Program description: procedures and files

High-Throughput GoMiner provides a choice of command line and web application interfaces. Here, we present a brief summary of both interfaces. The command line interface runs on Unix-based operating systems (including Mac OS X). Complete descriptions appear at the High-Throughput GoMiner website [3].

Both interfaces use the processing model envisioned in our original GoMiner article [1]:

1. A statistical operation identifies a set of changed genes.

2. The set of genes is listed in the GoMiner changed-gene file format.

3. GoMiner processes the total-and changed-gene files using a command line interface.

4. The results are exported from GoMiner and analyzed.

5. The user is notified which changed-gene files are of interest.

High-Throughput GoMiner generates two generic types of output. The first pertains to integrative results for all of the microarrays. The second pertains to each individual microarray.

In the integrative summary report, there are three entries for each microarray, corresponding to underexpressed, overexpressed, and total changed genes. The entries are sorted in descending order according to the potential interest of the result as indicated by the number of categories that satisfy a user-defined FDR. Integrative output files can be used to generate clustered image maps (CIMs) [5,6] showing 'significant categories' *versus *'microarrays.' By invoking a program that identifies transcription factor binding sites (R. Stephens, unpublished), High-Throughput GoMiner can also generate CIMs for 'transcription factor binding sites' *versus *'genes in a category' or 'transcription factor binding sites' *versus *'categories in a microarray'.

The web application version works with any browser. The user uploads a total-gene file and either a single changed-gene file or a zip file containing a set of changed-gene files. The user receives an email containing a hyperlink to a URL from which to download a compressed archive containing the results.

### Implementation

High-Throughput GoMiner is based on incorporation of command line GoMiner into a set of C Shell scripts. It is freely available and can be downloaded as a compressed tar file for use in a Unix-based environment. The scripts have been released under the GPL [7] open source license, so users are welcome to edit and extend them. The utilities used in the scripts (join, grep, gawk, sed, and curl) are commonly available in most Unix implementations or can be freely downloaded from sites such as the GNU Project [8]. The program was developed and tested on Mac OS X, Solaris, and Red Hat Linux. In our experience, there are slight variations among operating systems, computers, and versions of the Unix utilities. Porting between platforms might require minor user intervention, such as downloading gawk from the GNU Project rather than using awk.

**Figure 1:**
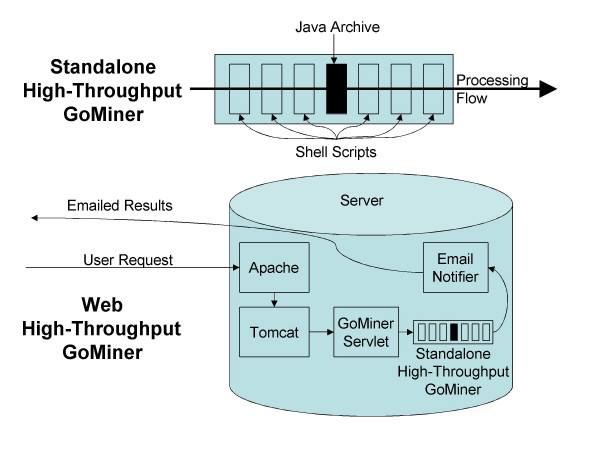
Schematic of stand-alone and web versions of High-Throughput GoMiner architecture and data flow.

The web application version of High-Throughput GoMiner (Figure 1) is implemented by using a simple Java servlet as a wrapper around the main scripts in the command-line version. The servlet manages the uploading of files, sets up an individual workspace for each request, and e-mails the user a URL for downloading results. To upload multiple changed-gene files, the user combines them into a single zip file. The web application version eliminates the minor manipulations described above for the command line version. It may take longer to complete requests because they are being executed in a shared environment, but the user interface is much simpler. The web version may be more suitable for the casual user who does not have the Unix background or resources to implement the command line version.

### Computational efficiency of High-Throughput GoMiner

High-Throughput GoMiner faces two daunting computational tasks: processing an arbitrary number of microarrays and performing many re-sampling instances in order to estimate the FDR. Each microarray typically requires hundreds of instances of re-sampling, and each instance requires the same computations as does analysis of the real data.

To make that computational burden manageable, we have developed a procedure that speeds up the processing time by several orders of magnitude relative to a naïve, brute force approach. At the heart of that procedure is the fact that the (real) total-gene file is used as both the (conceptual) total-and (conceptual) changed-gene files once, and the resulting gene-category export file is generated. The Unix 'join' utility is then applied to that file and to the (real) changed-gene files and re-sampled gene files *in lieu *of the much more time-consuming original GUI GoMiner process.

### The multiple comparisons problem in High-Throughput GoMiner: estimating a false discovery rate (FDR)

Most investigators use microarray results to decide what follow-up studies to do, rather than as definitive evidence. The goal of the statistical analysis is to provide the experimenter with a good list of candidate categories for follow-up. To decide whether or not to follow up a category that appears enriched in changed genes, the experimenter should know the statistical reliability of the apparent enrichment. However, without a multiple comparisons correction, some categories would appear enriched (have a low p-value in the Fisher test) simply by chance. To assess the significance of a particular category, we need to know the distribution of p-values that would occur by random chance. The expected number of false positives should be some manageable percentage (for example, less than 10%) of the categories selected. The percentage of false positives to be tolerated will generally depend on the relative costs of false positives and false negatives in whatever follow-up study is to be done. This way of framing the question leads us to specify the false discovery rate (FDR) for a set of categories, rather than significance level (p-value) for each category [9]. In practice High-Throughput GoMiner reports q-values for individual categories. The q-value (see below) represents the smallest false discovery rate at which that category would be classed as enriched.

For focus we will consider only 'biological_process' categories in the following discussion. Depleted categories are of less interest to most investigators than are enriched ones, so they will be ignored. Within a given category, the enrichment R_e _is given by

*R*_e _= (*n*_f_/*n*)/(*N*_f_/*N*)

where n_f _is the number of flagged genes within the particular category (*i.e*., genes whose expression levels are considered to be changed beyond a given threshold), n is the total number of genes within that same category, N_f _is the number of flagged genes on the entire microarray, and N is the total number of genes on the microarray.

The enrichment values are derived from categories of different sizes and do not have a common distribution. To assess the number of false positives, we need measures that are directly comparable. Fisher's exact p-values (for the one-tailed test) meet this need. See the original publication on GoMiner [1] for an extensive discussion of the statistical and conceptual bases for choosing the Fisher's exact test.

One way to address the multiple comparisons problem is to associate an individual measure of reliability for an ordered list of most enriched categories by specifying the FDR for each. Storey *et al*. [9] call this a q-value. The distinction between a q-value and a Fisher's Exact p-value as the two are used in the context of High-Throughput GoMiner is as follows:. The p-value, uncorrected for multiple comparisons, is a measure of the statistical significance of a single category. The q-value of a category is the FDR of the list of categories whose p-values are equal to or smaller than the p-value of that category.

To estimate the q-value for each category at each level of significance, we use a resampling algorithm. First, we select random samples of N_f _genes at each iteration and compute Fisher's exact test p-values for over-representation of the selected genes in all GO biological categories. After T resamplings, the q-value for the k-th most significant category is assessed as follows: We count the number of times that a Fisher's exact p-value less than or equal to the p-value, p_k_, of the k-th category, is found in the resampled data. Then, we divide by the number of resamplings:

m_k _= Σ_i=1,...,T _N(p < p_k _; i)/T,

where N(p < p_k _; i) refers to the number of p-values less than p_k _on resampling iteration i. Finally,

q_k _= m_k_/N_f_.

To ask how many permutation samples are needed before the q_k _values approximately reach their asymptotic values, we performed randomization studies (see 'Stability of Estimates of the False Discovery Rate' in Supplementary Materials [3]; [see Additional file 1]). Those studies indicate that the distribution of q-values for different categories in one resampling is usually not too different from the distribution of q-values for one category during many resamplings. Although T = 5000 is usual for permutation tests on microarray data, we found that, for data sets similar to those reported here for CVID, T = 1000 and T = 100 give essentially identical results. For any finite number of permutation samples, there is the possibility, of course, that one or more of the FDR estimates will be spuriously slightly below or slightly above the selected threshold value, but, as with ordinary p-values, slight deviations from the threshold shouldn't be over-interpreted. A menu in the web interface permits the user to select the appropriate number of resamplings. Only rarely does the ordering of categories by FDR differ from the ordering by Fisher's exact p-value. Thus, even though the FDR computation is only an approximation, it does not appreciably change which categories would have been given priority in the absence of multiple comparisons testing.

## Results

### Applying High-Throughput GoMiner to gene expression analysis of Clinical Common Variable Immunodeficiency (CVID)

We now illustrate the use of high-throughput GoMiner for interpretation of gene expression microarray data in a medical context, that of CVID. The input [see Additional file 2] and output [see Additional file 3] data are available as supplementary material.

### Background information on CVID

CVID is the most common symptomatic primary immunodeficiency disease, manifested by low levels of switched immunoglobulin isotypes (IgG, IgA, IgE) in the serum and by lack of humoral immune response to specific antigens [10]. It is a heterogeneous disease characterized by defects in humoral and cellular immunity [11]. The disease usually occurs in the second or third decade of life, often heralded by recurrent pyogenic infection [12]. CVID is associated with an increased incidence of autoimmune disease [13,14].

### High correlation of CVID and cancer

In one study, CVID patients were shown to have an 8-to 13-fold increased incidence of cancer overall, with a 438-fold increase in lymphoma for females [15]. Another, larger study showed an overall increased incidence of cancer of 1.8-fold, with the relative risks of stomach cancer and lymphoma at 10.3-and 12.1-fold, respectively [16]. The risk for non-Hodgkin's lymphoma over the period of 25 years has been estimated to be between 1.4% and 7.0% [17].

### Using microarray technology to study global gene expression in CVID

Microarray technology and analysis tools have made it possible to study global gene expression patterns in primary blood cells from CVID patients. To our knowledge this is the first such study, and it can contribute valuable information to what is currently known about the pathology and pathogenesis of the disease.

### Experimental methodology

Global gene expression patterns in twenty CVID patients are currently under study in our laboratory. One patient was selected for preliminary analysis and proof of concept for High-Throughput GoMiner. That patient had typical symptoms and laboratory findings, including repeated respiratory infections and low levels of all serum immunoglobulins.

Peripheral blood mononuclear cells (PBMC) from fresh blood were stimulated with CD3 and CD28 for 24 hours and used for RNA extraction. mRNA of the sample were amplified into aRNA and coupled with Cy3 or Cy5 fluorescent dye for microarray hybridization.

**Figure 2:**
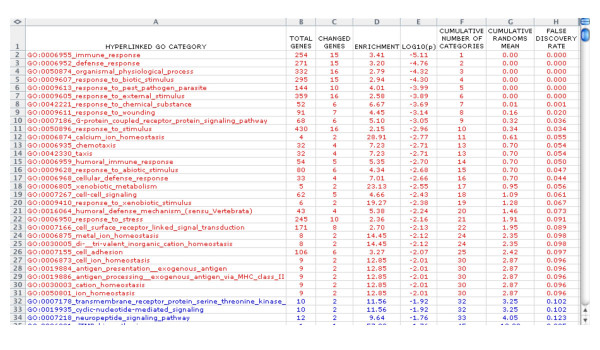
Screen shot of High-Throughput GoMiner results in Excel for GO categories enriched in genes with altered expression. The 30 GO categories with FDR = 0.10 are color-coded red; the other GO categories are color-coded blue.

**Figure 3:**
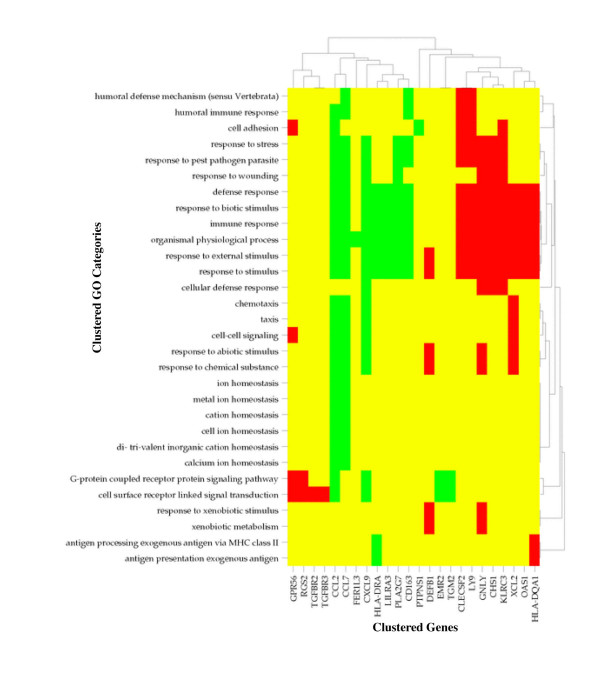
Clustered image map (CIM) [5,6] showing GO categories versus genes for genes with altered expression in a patient with CVID. Yellow indicates absence of the gene from the GO category. Red and green indicate over-and underexpressed genes, respectively. Clustering was performed with the Pearson correlation metric and average linkage algorithm. Instructions for using CIMminer to generate the CIMs in this paper are given in Supplementary Materials [see Additional file 5].

A detailed description of materials, methods, and data processing is provided in the Supplementary Materials [3]; [see Additional file 4]. An illustrative summary report (Figure 2) and clustered image map (CIM; Figure 3) are given in the main text (below).

### High-Throughput GoMiner identifies biologically-relevant categories

The CIM (Figure 3) serves as a fingerprint of the patient's molecular phenotype. The 24 genes with altered expression that caused these GO categories to be selected are shown at the bottom, and the categories are listed along the left-hand side. The largest category selected was 'response to external stimuli' (enrichment = 2.6-fold; p = 10^-3.9^; FDR = not detectable). 'Not detectable' means that the FDR was not distinguishable from zero given the number of randomizations used. See 'Summary Report' in the Supplementary Materials [3] for further details [see Additional file 6]. 'Response to external stimuli' contained 16 changed genes. All but one of those genes (DEFB1) were also in 'immune response' (enrichment = 3.4-fold; p = 10^-5.1^; FDR = not detectable). The Summary Report shows that, of the 254 genes in this GO category, 15 exhibited significant differences in expression level when the patient was compared with the normal controls. The categories 'cell surface receptor linked signal transduction' (enrichment = 2.7-fold; p = 10^-2.1^; FDR = 0.09), 'cell adhesion' (enrichment = 3.3-fold; p = 10^-2.1^; FDR = 0.10), and 'organismal physiological process' (enrichment = 2.8-fold; p = 10^-4.3^; FDR = not detectable), along with their child categories, contained the other changed genes.

The pathology of CVID in this patient was reflected by the GO categories with low values of FDR. CVID patients have defects in production of specific antibodies, and those defects may be associated with disturbed expression of 'immune/defense response genes' [18-22]. As indicated in Figure 3 and in Summary Report, the 'immune response' (enrichment = 3.4-fold; p = 10^-5.1^; FDR = not detectable), 'defense response' (enrichment = 3.2-fold; p = 10^-4.8^; FDR = not detectable), and related GO categories were prominent. The pathogenesis of CVID is not fully understood, but, from other primary immune deficiency diseases, we know that immunodeficiency may result from defects in 'antigen processing' (enrichment = 12.8-fold; p = 10^-2.0^; FDR = 0.10) [23,24], 'antigen presentation exogenous antigen' (enrichment = 12.8; p = 10^-2.0^; FDR = 0.10) [25], 'humoral immune responses' (enrichment = 5.4-fold; p = 10^-2.7^; FDR = 0.05) [26], and/or 'cell-cell signaling' (enrichment = 4.7-fold; p = 10^-2.4^; FDR = 0.06) [27-29]. Altered expression of genes in those categories may affect B cell activation, differentiation and maturation, and, ultimately, immunoglobulin production in CVID [30,31].

Several forms of signal transduction, not previously associated in the literature with CVID, were also enriched. Signal transduction plays an indispensable role in the immune response, and we suggest that signal transduction also plays an important role in CVID [27-29]. Defective B-or T-cell signaling can cause immunodeficiency, as can defective cytokine production or action [28,29]. Our microarray results show that several genes related to signal transduction (CCL2, CXCL9, EMR2, GPR56, RGS2, TGFBR2, RGFBR3, and TGM2 in the categories 'cell surface receptor linked signal transduction' (enrichment = 2.7-fold; p = 10^-2.1^; FDR = 0.09) and 'G-protein coupled receptor protein signaling pathway' (enrichment = 5.1-fold; p = 10^-3.1^; FDR = 0.04) are differentially expressed in the CVID patient. Full details of the roles of these genes in signal transduction, as well as the roles of all genes mentioned in this article, can be conveniently obtained *via *the hyperlinks to NCBI Entrez [32] in the Gene Category Report (Supplementary Materials [3]; [see Additional file 7]).

### High-Throughput GoMiner CIM facilitates grouping of closely-related categories into a single cluster

The 30 significant GO categories can be grouped into a smaller number of clusters, each of which contains several closely related categories. Grouping is desirable because it can remove the parent-child node redundancy inherent in the 'directed acyclic graph' (DAG) structure of GO. Removal of that redundancy effects a modest 'dimensionality reduction' and simplifies interpretation of the results *vis a vis *the disease phenotype. The CIM (Figure 3) of GO categories *versus *genes was used to create a tabulation (Table 1) of 7 clusters that result from grouping the 30 significant GO categories.

**Table 1:** Clusters Of Categories Derived From The CIM (Figure 3)

**Cluster Number**	**Cluster Name**	**Category**
1	Exogenous Antigen	antigen presentation exogenous antigen
		antigen processing exogenous antigen via MHC class II

2	Xenobiotic	xenobiotic metabolism
		response to xenobiotic stimulus

3a	Signaling	cell surface receptor linked signal transduction
		G-protein coupled receptor protein signaling pathway

4	Homeostasis	calcium ion homostasis
		di-tri-valent inorganic cation homeostasis
		cell ion homeostasis
		cation homeostasis
		metal ion homeostasis
		ion homeostasis

5a	Response	response to chemical substance
		response to abiotic stimulus

3b	Signaling	cell-cell signaling

6	Taxis	taxis
		chemotaxis

5b	Response	cellular defense response

5c	Response	response to stimulus
		response to external stimulus
		organismal physiological process
		immune response
		response to biotic stimulus
		defense response

5d	Response	response to wounding

5e	Response	response to pest pathogen parasite

		response to stress

7	Adhesion	cell adhesion

5f	Response	humoral immune response
		humoral defense mechansim

The CIM also permits detection of 'cross-talk' between GO categories that might at first appear to be unrelated. A number of examples can be found in Figure 3. For example, 'G-protein coupled receptor protein signaling pathway' and 'cell adhesion' both contain the changed genes GPR56 and CCL2. Detailed analysis of cross-talk can potentially provide an important systems biology interpretation of the particular set of significant GO categories in a disease state.

### High-Throughput GoMiner transcription factor binding site CIM can help to detect genomic regulatory networks

**Figure 4:**
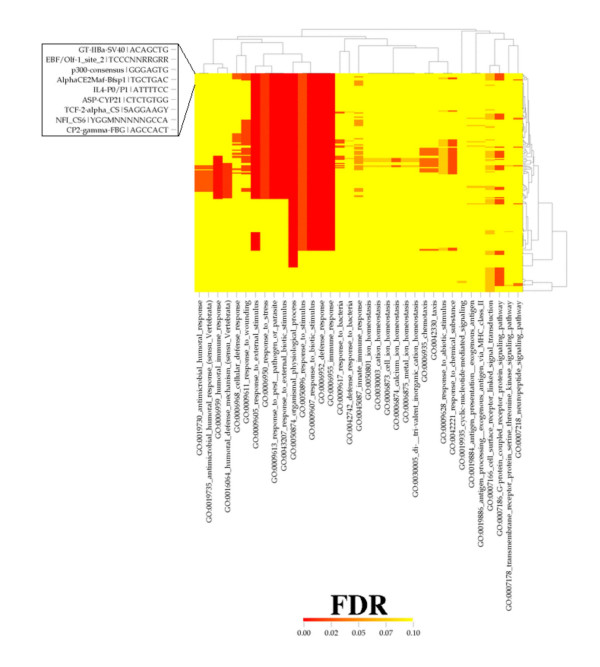
Clustered image map (CIM) 5,6 showing transcription factor binding sites *versus *GO categories in a patient with CVID. Red indicate FDR = 0.0, and yellow indicates FDR > 1.0 or a missing value. Clustering was performed with the Pearson correlation metric and average linkage algorithm. The inset is a blow-up of the first few transcription factor binding site names. A full-size version in which all the transcription factor binding site names are readable is available in Supplementary Materials [see Additional file 8]. There are 35 rather than 30 GO categories because this result was computed with a more recent version of the GO Consortium database.

Figure 4 indicates the richness of information available for inference of genomic regulatory networks from a CIM for transcription factor binding site vs. GO category. A full-size version in which all the transcription factor binding site names are readable is available in the Supplementary Materials [see Additional file 8]. Among the numerous relationships that can be mined by a systematic analysis of this novel type of CIM is a set of transcription factors (Table 2) that co-regulate the changed genes in the GO category 'G-protein coupled receptor protein signaling pathway' and a large core of 'response' categories.

**Table 2:** Names and Consensus Sequences for Transcription Factors that Co-Regulate the Changed Genes in the GO Category 'G-protein Coupled Receptor Protein Signaling Pathway' and a Large Core of 'Response' Categories (Figure 4 and Supplementary Materials [see Additional file 8])

**Name**	**Consensus**
AP-1-IL-3	TGAGTCA
AP-1-involucrin-H2	TGCCTCA
ASP-CYP21	CTCTGTGG
AlphaCE2Maf-Bfsp1	TGCTGAC
B2_RS	TCCTATCA
CP2-consensus	GCNMNANCMAG
CPSI-B1	TCTCCCA
EBF/Olf-1_site_2	TCCCNNRRGRR
EBV-ZRE2	TGAGCAA
FHX-type-A-CS	WMARYAAAYA
GAGA_box/CT_element	AGAGARRRR
GH-CSE2	AATAAAT
GRE_CS7	WCTGWTCT
GRE_CS8	AGAWCAGW
GT-2B_RS	CCAGCTG
GT-IIBa-SV40	ACAGCTG
HNF5-erk1	TATTTGT
HiNF-Ahist	AGAAATG
IL4-P0/P1	ATTTTCC
Initiator_CS	CTCANTCT
MEF-2-consensus	YTWWAAATAR
MEF-2_CS	YTAWAAATAR
NF-Y-consensus	BVDCCAATVVVVD
PuF_RS	GGGTGGG
RadLV-core	TGTGGTCA
Runx_CS	AACCACA
Six5_CS	TCARRTTNC
Sp1-VGF_1	AGGGAGG
TCF-2-alpha_CS	SAGGAAGY
TCR-beta-site-6	AATACAA
TRE.1	TGACTCA
c-Myc_RS1	TCTCTTA
c-mos_DS3	GTTTTAA
delta-rpL7	GGAGGCTG
forkhead_CS	WAARYAAAYW
p300-consensus	GGGAGTG

A detailed discussion of differential expression of individual genes is provided in Supplementary Materials [3]; [see Additional file 9].

### Correlation of GO categories and disease phenotype

The GO categories and genes identified by High-Throughput GoMiner are shown in Figure 3 and in Gene Category Report. The categories and genes correlate well with the disease's phenotype. Thus, High-Throughput GoMiner can integrate information from entire gene expression microarray studies into a coherent picture of biological process gene category and disease phenotype at the molecular level. It provides information with which researchers can develop new hypotheses or explore potential therapeutic targets.

### Applying High-Throughput GoMiner to gene expression analysis of schistosomiasis

**Figure 5:**
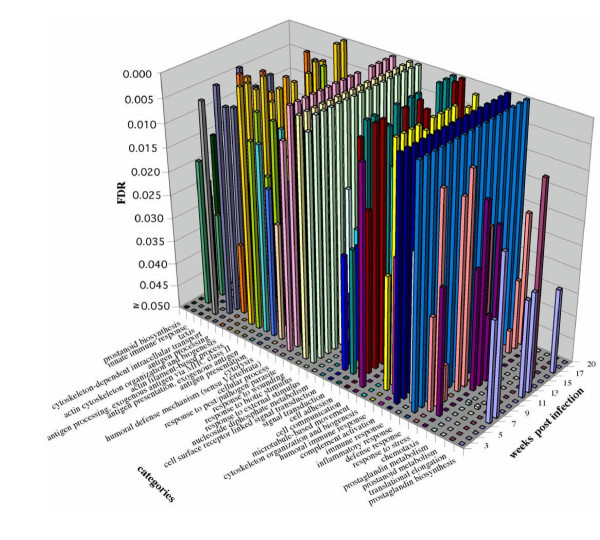
Time series for GO categories with low FDR for overexpressed genes. The data were obtained from a study of schistosomiasis in a murine model [37-40] over the course of 20 weeks after infection. 3D bar graph visualization in Excel. (*Elnekave et al*, in preparation).

**Figure 6:**
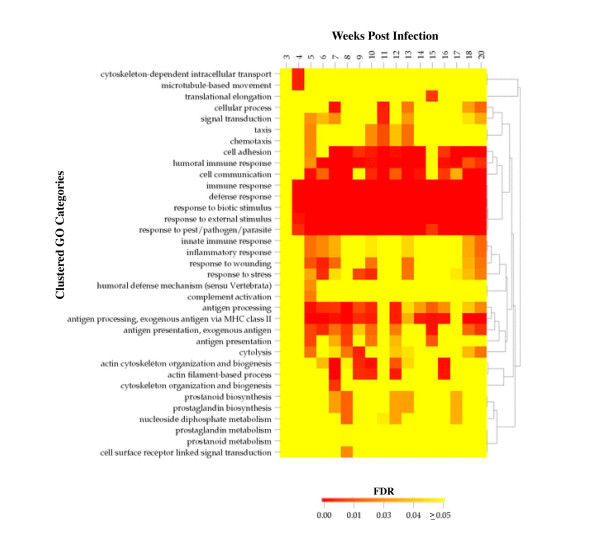
CIM [5,6] with hierarchically clustered categories (Pearson correlation, average linkage clustering) versus time (Elnekave *et al*., in preparation).

To highlight how High-Throughput GoMiner can be used to integrate time series data, we consider briefly an example from preliminary analysis of gene expression in schistosomiasis (Elnekave *et al*., in preparation). Schistosomiasis is associated with bladder cancer in third-world countries [33-36]. Figure 5 shows the GO categories with low FDR for overexpressed genes in the form of a 3-D bar graph in Excel. Figure 6 shows a clustered image map (CIM) generated using CIMminer [6] to show the time course.

## Discussion

### Comparison of High-Throughput GoMiner with related programs

While this work was in progress, a number of tools for GO analysis of microarray data have become available. Included are EASE [41], FatiGO [42], FunSpec [43], GoSurfer [44,45], GO::TermFinder [46,47], Onto-Express [48], and Ontology Traverser [49]. However, none of them have the central integrative features that characterize High-Throughput GoMiner. Of those tools, only GO::TermFinder permits batch processing. Since the others have been reviewed previously and since the main features of High-Throughput GoMiner relative to the original GoMiner package derive from batch processing, we will confine the present discussion to a comparison between GO::TermFinder and High-Throughput GoMiner.

High-Throughput GoMiner appears to offer all of the functionality of GO::TermFinder, as well as additional important features. In an earlier section, entitled 'Significant Advances Relative to the Original Version of GoMiner,' we detailed the scientific value-added and usability features of High-Throughput GoMiner *vis a vis *the original GoMiner. Those same scientific value-added and usability features distinguish High-Throughput GoMiner from GO::TermFinder. Because the detailed description can be found in the above-mentioned section and technical details can be found in the Output Files page of the High-Throughout GoMiner web site [3], we confine ourselves here to listing the scientific value-added and usability features of High-Throughput GoMiner that are not present in GO::TermFinder:

1. Integration of information across multiple microarrays in a study: CIMs (*e.g*., Figure 5, 6).

2. Integration across multiple microarrays: category|gene-disease export files.

3. CIMs of genes with altered expression *versus *significant GO categories (Figure 3).

4. Annotation of genes and GO categories with transcription factor binding site information (Figure 4).

5. Prioritization of microarrays in a study on the basis of the number of categories that are statistically significantly enriched.

There are also a number of usability features that distinguish High-Throughput GoMiner from GO::TermFinder:

6. Greater simplicity in the running of High-Throughput GoMiner. The GO::TermFinder batch processing feature [50] is implemented through the 'analyze.pl' module. The implementation requires that the user supply both an annotation file and an ontology file, obtained from the GO Consortium web site. In contrast, High-Throughput GoMiner accesses the annotation and ontology information through a database that we maintain on our server, so the low-level implementation details are transparent to the user.

7. Greater generalization of annotations in High-Throughput GoMiner. The default mode of High-Throughput GoMiner includes all annotations in the GO Consortium database, with the option to restrict the annotations to any arbitrary combination of annotation sources.

8. Greater control over species selection for High-Throughput GoMiner. The default mode of High-Throughput GoMiner permits restricting the query to any single species *(e.g.,* mouse or human) or to any combination of species represented within the GO Consortium database. That is a functionally important type of flexibility.

9. Recognition of HUGO gene names by High-Throughput GoMiner. The High-Throughput GoMiner database can recognize HUGO names as well as any of the other identifier types provided by the GO Consortium database. The ability to recognize HUGO names is not an inherent feature of the annotation provided by the GO Consortium, so users of GO::TermFinder are not able to access HUGO names in their queries.

10. Platform-independence of the Web Interface Version of High-Throughput GoMiner. The Web Interface Version of High-Throughput GoMiner is platform-independent because it is a web server application. In contrast, a Unix environment is required to use the batch processing capability of GO::TermFinder [51].

11. Output formatting differs between High-Throughput GoMiner and GO::TermFinder. The latter appears to present output in a list format, whereas several modes of output (at the level of either individual microarray or integration of all microarrays) are available with High-Throughput GoMiner.

In summary, of the tools available for using GO to interpret microarray (or analogous) data, only GO::TermFinder and High-Throughput GoMiner offer batch-processing capability. GoMiner, in addition, provides tools and visualizations for *integrating *information from the batch of microarrays and for relating them to transcription factor binding sites and regulatory networks. Because of additional scientific and usability characteristics of High-Throughput GoMiner, it is particularly well suited to the needs of the molecular biology, genomics, and proteomics communities.

## Acknowledgements

The research was supported [in part] by the Intramural Research Program of the N1H, National Cancer Institute, Center for Cancer Research.

## Conclusion

High-Throughput GoMiner efficiently performs the computationally challenging task of automated batch-processing of an arbitrary number of microarrays (or other conceptually similar sets of large data sets). To our knowledge, it is the first resource for integration of high-throughput analyses of multiple microarrays. The automatically generated output files permit visualization of time series data in a 3-D bar chart in Excel or as a hierarchically clustered image map (CIM) of the interesting GO categories in relation to expression (or transcription factor binding sites).

As a proof of concept, we used High-Throughput GoMiner to analyze the results of a microarray study of differences in gene expression between a patient with CVID and normal controls. Because the phenotypes of immunodeficiency diseases have been well described, we were able to demonstrate that the GO categories found by High-Throughput GoMiner were those that were expected (*e.g*., 'immune response'). Since this was the first global gene expression study of CVID, the analyses using High-Throughput GoMiner have provided new information on biological process categories and specific genes in the disease. For example, most of the differentially expressed genes were found in signal transduction categories. Signal transduction had not previously been reported to play a role in CVID. High-Throughput GoMiner thus has the potential to generate new biomedical hypotheses and identify new targets for research.

We have tried to make High-Throughput GoMiner as flexible as possible by providing both command line and web server versions. The command line version provides faster calculation and the potential for highly parallel processing; the web server version provides transparency and ease of use. The next phases of genomics and proteomics will impose increasing demands for flexible, large-scale, automated information processing. We see High-Throughput GoMiner as a key resource for addressing that challenge.

## Availability and requirements

Project name: High-Throughput GoMiner; Project home page: ; Operating system(s): web version is platform independent, command line version requires Unix; Programming language: java and Unix C shell; Other requirements: detailed on web site and on documentation packaged in command line version download; License: GNU GPL; Restrictions to use by non-academics: none.

## Supplementary Material

Additional File 1 Stability of Estimates of the False Discovery Rate Click here for file

Additional File 2 Expression Data Click here for file

Additional File 3 Output Files Generated from High-Throughput GoMiner Click here for file

Additional File 4 Methodology Description Click here for file

Additional File 6 Summary Report Click here for file

Additional File 7 Gene Category Report Click here for file

Additional File 8 CIM of Transcription Factors versus GO Categories Click here for file

Additional File 9 Discussion of Results Click here for file

Additional File 5 Instructions for Generating the CIMs in the Manuscript Click here for file
