# Supplementary figures and images for: High-Throughput GoMiner, an 'industrial-strength' integrative gene ontology tool for interpretation of multiple-microarray experiments, with application to studies of Common Variable Immune Deficiency (CVID)
Source: BMC Bioinformatics. 2005 Jul 5;6:168. doi: 10.1186/1471-2105-6-168 (PMC1190154; doi:10.1186/1471-2105-6-168)

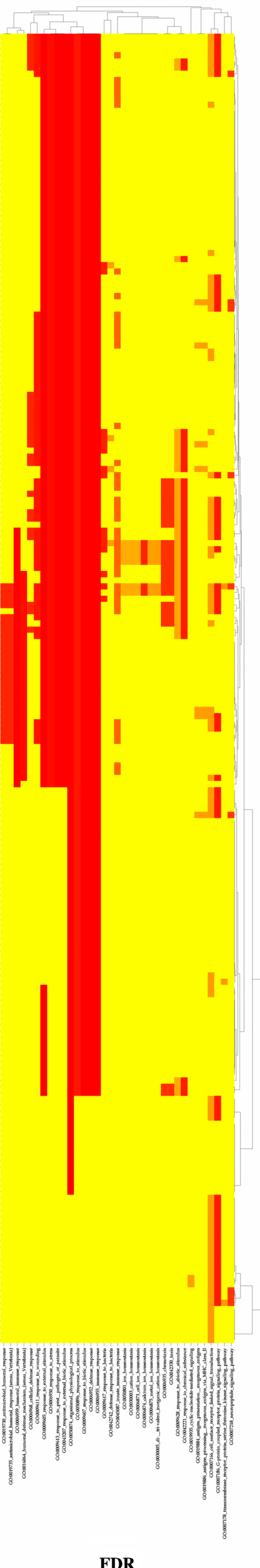

FDR

0.00 0.02 0.05 0.07 0.10

Supplement: Additional File 8 — CIM of Transcription Factors versus GO Categories [file 1471-2105-6-168-S8.pdf]
