# Supplementary material for: High-Throughput GoMiner, an 'industrial-strength' integrative gene ontology tool for interpretation of multiple-microarray experiments, with application to studies of Common Variable Immune Deficiency (CVID)
Source: BMC Bioinformatics. 2005 Jul 5;6:168. doi: 10.1186/1471-2105-6-168 (PMC1190154; doi:10.1186/1471-2105-6-168)
Supplement: Additional File 5 — Instructions for Generating the CIMs in the Manuscript [file 1471-2105-6-168-S5.pdf]

Instructions for Using CIMminer with High-Throughput GoMiner Output Files

Barry Zeeberg
barry@discover.nci.nih.gov
September 30, 2004

1. Overview
    - The CIMminer user interface is in the process of reorganization. In the meantime, we are providing specific instructions to users of High-Throughput GoMiner. The purpose of these instructions is to facilitate construction of clustered image maps (CIMs) that are similar to those shown in figures in the High-Throughput GoMiner manuscript, as well as other CIMs that can be constructed from High-Throughput GoMiner output files.

2. General Observations
    - Internet Explorer is the preferred browser for CIMminer.
    - Certain platforms and browsers can cause a problem during the upload of the input file unless the input file name ends in ".txt". This problem has been noted in particular on Mac OS X (10.2.8) using Internet Explorer 5.2.3.
    - Another potential problem occurs if all of the rows of the input file do not have exactly the same number of field delimiters (*i.e.*, tabs). We have taken pains to avoid introducing this problem in the output files of High-Throughput GoMiner.
    - The error messages that result from these two conditions are not informative, and can, in fact, be misleading.
    - Another error message that is not informative can result from an attempt to use the correlation metric if there is a row or column all of whose entries are identical.
    - These and other usability issues are currently being addressed.

3. Specific Instructions for Figure 3 of the High-Throughput GoMiner manuscript
    - We refer to CIMs similar to Figure 3 as "trinary" because they contain exactly three values. In particular, the values are -1, 0, and 1.
    - This is the type of file that is described on the High-Throughput GoMiner web site under the "Output Files" tab as "*CHANGEDGENESFILE*.change.gce.updown.CIM".
    - Steps - steps (b) through (d) should be performed simultaneously
        a. Clustering

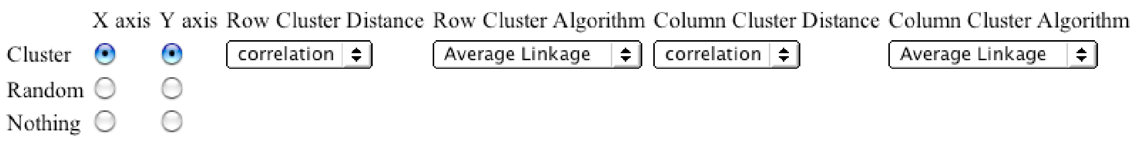

b. Colors

Minimum value	Left mid-point	Right mid-point	Maximum value
(•) 0,1,0	( ) 1,1,0	( ) 1,1,0	( ) 1,0,0

c. Page Layout

Rotation	0 degree	Transposition of data	transpose
Height of CIM*	550	Width of CIM*	450
Width of legend*	144	Height of legend*	4.5

d. Axes

Axes

TOP	BOTTOM
Draw: Cluster tree, N/A	Draw: Label, Facing to the left
**LEFT**	**RIGHT**
Draw: Label, Facing up	Draw: Cluster tree, N/A
**Font size for all labels**	10

e. Save CIM as .gif

Right-click to reveal menu and select “Download Image to Disk”

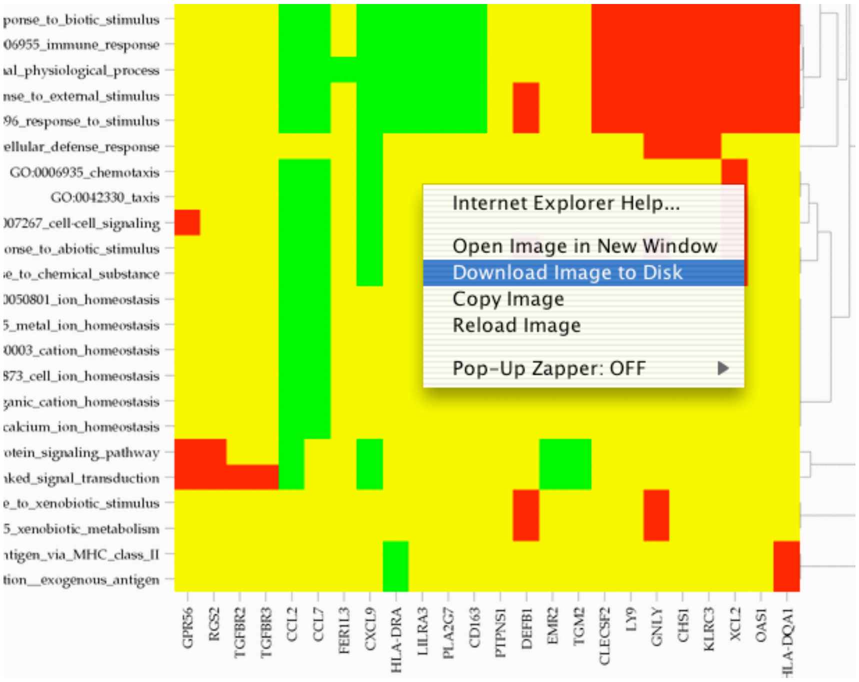

4. Specific Instructions for Figure 4 of the High-Throughput GoMiner manuscript
   - We refer to CIMs similar to Figure 4 as "continuous" because the entries can assume any floating point values. In this particular instance, the values are all non-negative.
   - This is the type of file that is described on the High-Throughput GoMiner web site under the "Output Files" tab as "total.txt.change.series.CIM".
   - Steps - steps (b) through (e) should be performed simultaneously
     a. Clustering

	X axis	Y axis	Row Cluster Distance	Row Cluster Algorithm	Column Cluster Distance	Column Cluster Algorithm
Cluster	◉	○	correlation	Average Linkage	correlation	Average Linkage
Random	○	○				
Nothing	○	◉				

b. Colors

Minimum value	Left mid-point	Right mid-point	Maximum value
◉ 1,1,0	○ 1,.5,0	○ 1,.5,0	○ 1,0,0

c. Binning

Binning method	Asymmetric interval

d. Page Layout

Rotation	0 degree	Transposition of data	transpose
Height of CIM*	550	Width of CIM*	450
Width of legend*	144	Height of legend*	4.5

e. Axes

**TOP**	**BOTTOM**
Draw: Label / Facing to the left	Draw: Nothing / N/A
**LEFT**	**RIGHT**
Draw: Label / Facing up	Draw: Cluster tree / N/A
**Font size for all labels**	10

f. Save CIM as .gif

Right-click to reveal menu and select “Download Image to Disk”

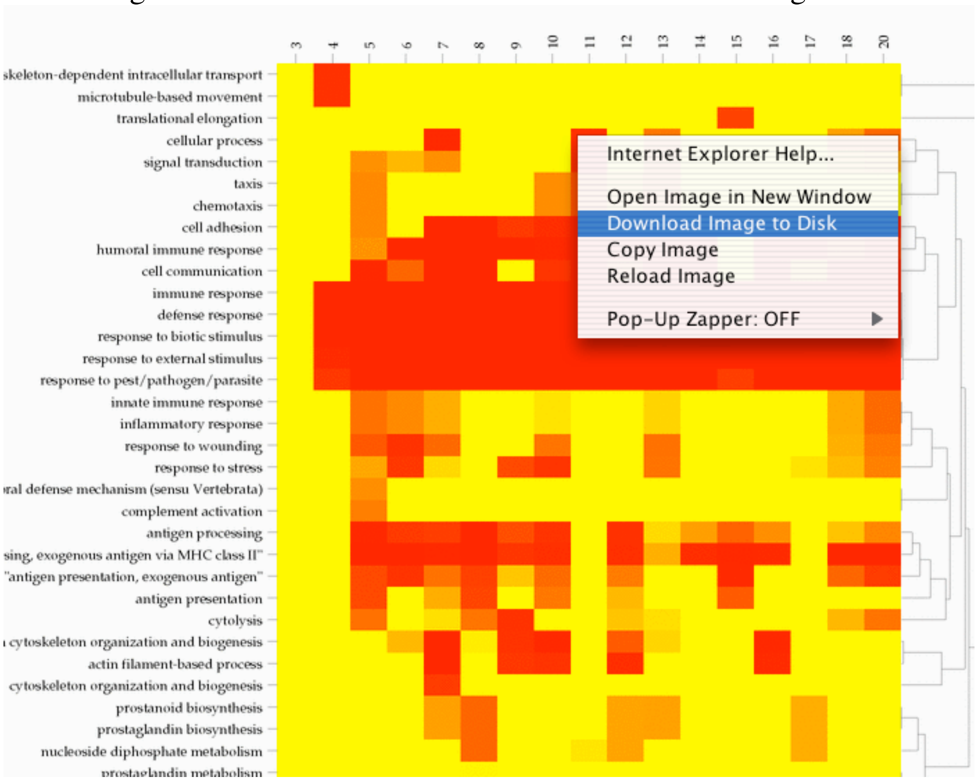

Pitfalls

• If you get the error message: "There was an error processing your files in R, The cluster distance you selected is not defined for your dataset. For example, you selected "binary" distance, your dataset is not discrete (character) data," be sure your dataset is appropriate for the distance metric you selected. Examine your data and remove the offending row or column, and try to generate the CIM again.

• If you get the error message: "Your datafile has an inconsistent number of tab separated values per line," a possible culprit could be extra tabs at the end of your data files. You should not get this error when processing data directly from High-Throughput GoMiner, but it is possible to accidentally introduce this error if you manually edit the cluster data file.
